# Retraction Note: Anti-cancer effects of grailsine-al-glycoside isolated from Rhizoma Sparganii

**DOI:** 10.1186/s12906-017-1767-3

**Published:** 2017-05-11

**Authors:** Jun-Wu Zhang, Ya-Hui Wei

**Affiliations:** 1 College of Life Science, Northwest University, Key Laboratory of Resource Biology and Biotechnology in Western, Xi’an, China; 2 College of Pharmacy, Shaanxi University of Chinese Medicine, Xi’an, China

## Erratum

This article [1] has been retracted by the Editor. Fig. 1, which was part of a doctoral dissertation by Jie Sun (Northwest University) and previously published [2], as well as Figs. 3, 4 and 5 from the same dissertation, were modified and reproduced in this article without permission. In addition, the authors informed the Editor that they are unable to reproduce key data presented in the article. The findings of this study are therefore unreliable. The Editor has been unable to confirm with Northwest University whether an institutional investigation has taken place. Both of the authors agree with this retraction.
